# Suture‐augmented primary anterior cruciate ligament repair with internal brace shows acceptable re‐rupture rates, favourable outcomes and high return‐to‐sport rates: A systematic review

**DOI:** 10.1002/jeo2.70495

**Published:** 2025-11-05

**Authors:** Alessandro Carrozzo, Émilie Bérard, Valerio Nasso, Edoardo Monaco, Jonathan Rioual, Régis Pailhe, Etienne Cavaignac

**Affiliations:** ^1^ Dipartimento di Scienze della Vita, della Salute e delle Professioni Sanitarie Università degli Studi “Link Campus University” Rome Italy; ^2^ Department of Clinical Epidemiology and Public Health CERPOP, INSERM‐University of Toulouse III, Toulouse University Hospital (CHU) Toulouse France; ^3^ Department of Orthopaedic Surgery and Traumatology, AOU Sant′Andrea La Sapienza University of Rome Rome Italy; ^4^ Research Methodological Support Unit (USMR), Department of Clinical Epidemiology and Public Health Toulouse University Hospital (CHU) Toulouse France; ^5^ Clinique Aguilera Ramsay Santé Biarritz Biarritz France; ^6^ Department of Orthopaedic Surgery Hôpital Pierre Paul Riquet, CHU de Toulouse Toulouse France

**Keywords:** ACL repair, internal brace, patient‐reported outcomes, re‐rupture rates, suture augmentation

## Abstract

**Purpose:**

To describe the safety, efficacy and clinical outcomes of suture‐augmented primary anterior cruciate ligament (ACL) repair with internal bracing.

**Methods:**

A systematic review was conducted following Preferred Reporting Items for Systematic Reviews and Meta‐Analyses guidelines; the protocol was registered in the PROSPERO database (registration number: CRD42024573962). A comprehensive search of the PubMed, Embase and Cochrane Library databases up to August 2024 was conducted to identify studies reporting clinical outcomes of suture‐augmented ACL repair. Inclusion criteria were a minimum follow‐up of 2 years, reporting of re‐rupture rates together with complications, or functional outcome scores such as International Knee Documentation Committee (IKDC), Knee injury and Osteoarthritis Outcome Score (KOOS), Lysholm or Tegner activity scale.

**Results:**

Thirteen studies involving 671 patients met the eligibility criteria and were included. The ACL re‐rupture rate was 10.6% (95% confidence interval [CI]: 7.4–14.2), and the rate of reoperations unrelated to ACL re‐rupture (primarily for hardware removal) was 3.2% (95% CI: 0.9–6.5). The pooled data showed a mean time to reinjury of 1.2 years (95% CI: 1–1.3), and failure occurred at a mean patient age of 19.8 years (95% CI: 14.8–24.8). The mean return to sport was 9.5 months (95% CI: 5.7–13.3). Patient‐reported outcome measures included IKDC with a post‐operative mean score of 86.9 (95% CI: 83.6–90.1) and KOOS total score of 85 (95% CI: 82.1–88). KT‐1000 laxity measurements revealed a mean side‐to‐side difference of 0.9 mm (95% CI: 0.5–1.3).

**Conclusion:**

Suture‐augmented primary ACL repair with internal brace shows acceptable clinical results, encouraging functional recovery and re‐rupture rates. Internal bracing is not associated with a high rate of complications, suggesting that the technique is safe and appropriate for use in carefully selected patients. However, heterogeneity in the existing studies means that additional high‐quality, long‐term comparative studies are needed to clarify the indications and reliability of this procedure.

**Level of Evidence:**

Level IV, systematic review of studies with LoE I–IV.

**Abbreviations**
ACL: anterior cruciate ligament
CI: confidence interval
IKDC: International Knee Documentation Committee
KOOS: Knee injury and Osteoarthritis Outcome Score
LoE: level of evidence
MUA: mobilization under anaesthesia
PRISMA: Preferred Reporting Items for Systematic Reviews and Meta‐Analyses
PROMs: patient‐reported outcome measures
PROSPERO: International Prospective Register of Systematic Reviews
ROBINS‐I: Risk of Bias in Non‐randomized Studies‐Interventions
RTS: return to sport
SA: suture‐augmented
SANE: single assessment numeric evaluation
SD: standard deviation
VAS: visual analogue scale
WOMAC: Western Ontario and McMaster Universities Osteoarthritis Index

## INTRODUCTION

In the early 1900s, Sir Mayo Robson first described primary anterior cruciate ligament (ACL) repair and established it as the first surgical procedure for ACL‐deficient knees [38]. During the 1970s and 1980s, this procedure became the standard of care, with initially favourable short‐term results [12, 29, 33]. However, medium‐ and long‐term follow‐up revealed significant rates of knee instability, stiffness and overall unpredictability [5, 10, 13, 28]. This led to the procedure being abandoned and a subsequent shift towards ACL reconstruction, which offered more reliable outcomes and allowed earlier rehabilitation and return to activities [30, 40].

Nevertheless, in recent years, there has been a resurgence of interest in ACL repair due to its theoretical advantages over ACL reconstruction [3, 17, 37]. These include improved post‐operative proprioception due to preservation of the native ligament, a less invasive and faster technique that avoids the morbidity associated with graft harvest and requires smaller tunnels or sockets, and faster return to function allowing for earlier return to sport (RTS) [14].

Various techniques for ACL repair have been proposed. One of these new techniques involves augmenting the ACL repair with an ultra‐high‐molecular‐weight polyethylene/polyester tape coated with collagen, also known as an Internal Brace [44]. Suture‐augmented (SA) ACL primary repair seems to offer significant advantages during the ACL healing process, due to enhanced tensile strength and reduced elongation risks, ultimately leading to lower re‐rupture rates [32]. While promising, there is insufficient high‐quality evidence to definitively establish the significant advantages of internal bracing over other techniques.

The aim of this systematic review was to describe the safety and efficacy of SA primary ACL repair with internal bracing and to summarize the current evidence on the clinical outcomes. The hypothesis was that this technique is safe and effective, providing acceptable re‐rupture rates and favourable functional outcomes in carefully selected patients.

## METHODS

### Study design

A systematic review of the literature was conducted to investigate clinical outcomes after ACL repair with internal brace augmentation. The study was conducted according to Preferred Reporting Items for Systematic Reviews and Meta‐Analyses (PRISMA) guidelines [34]. The systematic review protocol was registered with the PROSPERO (International Prospective Register of Systematic Reviews) database (registration number: CRD42024573962).

### Data sources, search strategies and eligibility for inclusion

A comprehensive literature search was conducted using the PubMed, Embase and Cochrane Library databases up to 30 August 2024. The search strategy combined controlled vocabulary (MeSH terms) with free‐text terms relating to primary repair of the ACL, internal bracing and suture augmentation. Filters were applied to limit the results to human studies published in English (Appendix S1). Studies focusing on SA ACL primary repair with internal bracing were selected. Studies were included if they met the following criteria: included patients underwent SA primary ACL repair with or without comparison to patients who underwent ACL reconstruction; reported at least one of the following outcome measures: re‐rupture rate, complications, RTS, patient‐reported outcome scores (PROMs) such as the International Knee Documentation Committee (IKDC) scores, Knee Injury and Osteoarthritis Outcome Score (KOOS), visual analogue scale (VAS) for pain, Tegner activity scale, Lysholm score, SANE score, MARX score, Western Ontario and McMaster Universities Osteoarthritis Index (WOMAC) or KT‐1000 arthrometer measurements; had a minimum follow‐up of 2 years. Studies were excluded if were non‐clinical (biomechanics, cadaver or animal studies); reviews, case reports, conference abstracts; clinical articles from the pre‐arthroscopy era; articles written in a language other than English; repair techniques other than direct suture with the addition of an internal brace; multiligament lesions (other than the anterolateral ligament); populations with a history of ipsilateral knee pathology; follow‐up of less than 2 years; studies performed exclusively in paediatric populations; studies that did not report re‐rupture rates.

### Study selection

Two investigators independently screened titles and abstracts for eligibility and then assessed the full text (A.C. and V.N.). Each article was reviewed for relevance, and the references of chosen articles were checked to identify additional relevant studies. Disagreements were resolved by discussion with the senior author (E.C.).

A quality assessment of the methodological rigour of the included studies was performed using the Risk of Bias in Non‐randomized Studies‐Interventions (ROBINS‐I) tool, and the level of evidence (LoE) was assigned according to the Oxford Centre for Evidence‐Based Medicine [9].

### Data extraction and collection

Data were extracted independently by two investigators using a standardized database. Extracted information consisted of study design, size of the study population, demographics, surgical technique, concomitant procedures, outcomes, complications and follow‐up time. Demographic data were the sex ratio and the mean patient age. Clinical outcomes were the PROMs, assessment of knee stability (side‐to‐side anterior‐posterior laxity difference) and RTS. The Tegner activity scale was used to assess the level of sport achieved post‐operatively.

The primary outcome was the re‐rupture rate following SA ACL primary repair. Secondary outcomes included non‐rupture related complications, RTS, functional outcome scores, knee laxity measures and time to reinjury (i.e., the time elapsed between surgery and re‐rupture).

### Statistical analysis

To describe the results in the ACL repair group, qualitative parameters were summarized by presenting the number of events and the total number of subjects per study. The global proportion was then calculated, along with its 95% confidence intervals (CIs), using the Freeman–Tukey transformation [18], which is recommended to stabilize variance, especially when estimated proportions are close to 0 or 1. For quantitative parameters, the mean, standard deviation (SD) and total number of subjects for each study were reported, and the overall mean was calculated with its corresponding 95% CI. We chose to use a random‐effects model. Studies were then weighted using inverse variance weighting.

## RESULTS

### Study selection and characteristics

**Figure 1:**
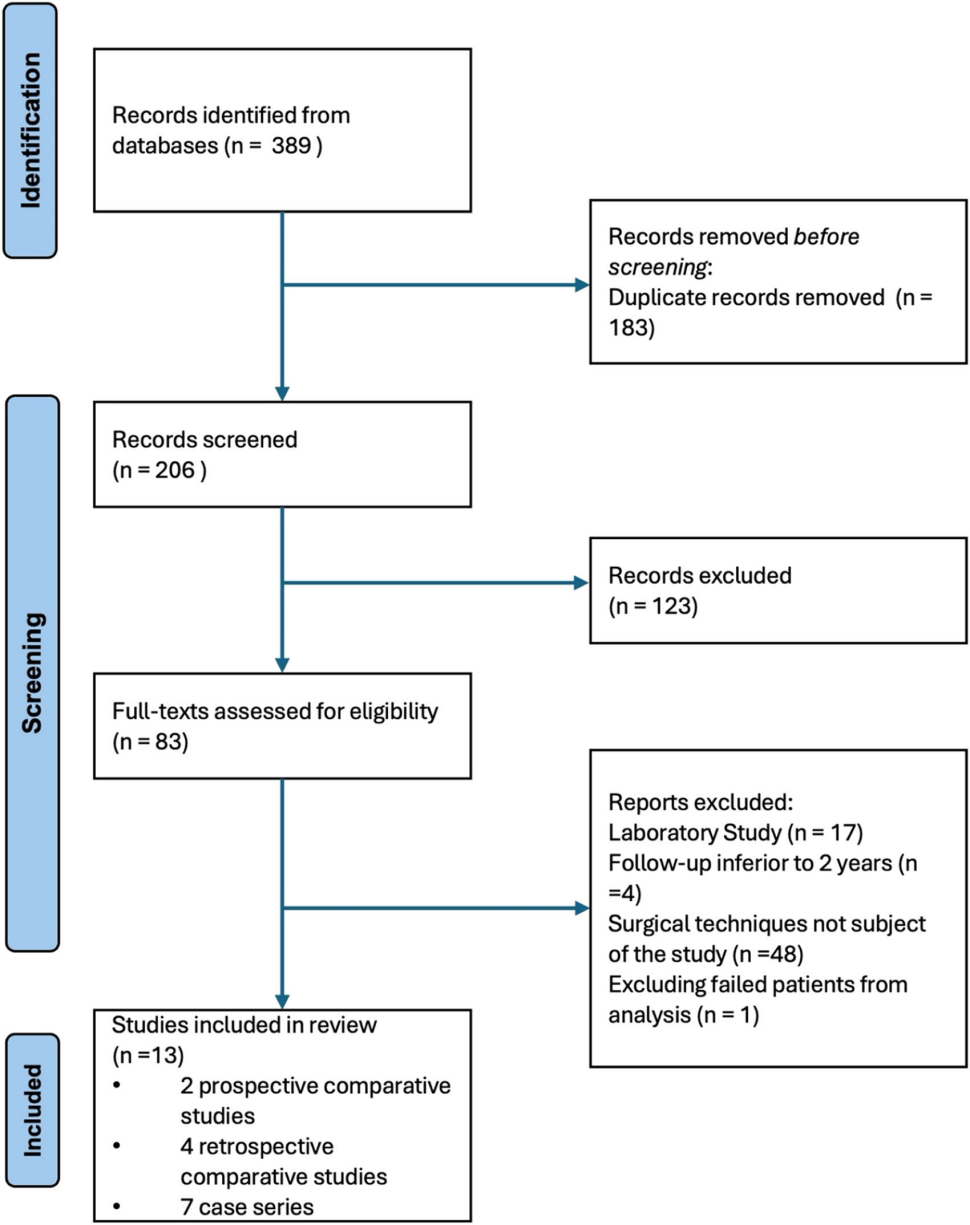
PRISMA flow diagram. PRISMA, Preferred Reporting Items for Systematic Reviews and Meta‐Analyses.

A total of 389 records were initially identified through database searches. After removing duplicate entries and applying the inclusion criteria, 83 full‐text articles were then screened for eligibility. In the end, 13 studies met the eligibility criteria and were included in the systematic review (Figure 1). The included studies varied in design from prospective comparative studies to case series (Table 1).

**Table 1:** Summary of included studies on suture‐augmented primary ACL repair with internal bracing.

Title of the study	Author year	Study design	Level of evidence
Primary Anterior Cruciate Ligament Repair Using Suture Tape Augmentation: A Case Series of 29 Patients With Minimum 2‐Year Follow‐Up [4]	Burton 2021	Case series	IV
Suture‐Augmented Anterior Cruciate Ligament Repair for Proximal Avulsion or High‐Grade Partial Tears Shows Similar Side‐to‐Side Difference and No Clinical Differences at Two Years Versus Conventional Anterior Cruciate Ligament Reconstruction for Mid‐Substance Tears or Poor Anterior Cruciate Ligament Tissue Quality [7]	Douoguih 2024	Prospective cohort study	II
Satisfactory patient‐reported outcomes at 5 years following primary repair with suture tape augmentation for proximal anterior cruciate ligament tears [23]	Hopper Aithie 2022	Case series	IV
Failure Rates After Anterior Cruciate Ligament Repair With Suture Tape Augmentation in an Active‐Duty Military Population [6]	Cruz 2023	Prospective cohort study	II
Combined Anterior Cruciate Ligament Repair and Anterolateral Ligament Internal Brace Augmentation Minimum 2‐Year Patient‐Reported Outcome Measures [24]	Hopper 2020	Case series	IV
Anterior Cruciate Ligament Repair with Suture Augmentation for Proximal Avulsion Injuries [8]	Douoguih 2020	Case series	IV
Anterior cruciate ligament repair with Independent Suture Tape Reinforcement: a case series with 2‐year follow‐up [22]	Heusdens 2019	Case series	IV
Arthroscopic primary repair of proximal anterior cruciate ligament tears: outcomes of the first 56 consecutive patients and the role of additional internal bracing [26]	Jonkergouw 2019	Retrospective cohort study	III
Suture tape augmentation ACL repair, stable knee, and favorable PROMs, but a re‐rupture rate of 11% within 2 years [21]	Heusdens 2021	Case series	IV
Promising functional outcomes following anterior cruciate ligament repair with suture augmentation [39]	Schneider 2022	Case series	IV
Comparable rates of secondary surgery between anterior cruciate ligament repair with suture tape augmentation and anterior cruciate ligament reconstruction [25]	Hopper Wilson 2022	Retrospective cohort study	III
The Minimal Clinically Important Difference, Patient Acceptable Symptom State, and Clinical Outcomes of Anterior Cruciate Ligament Repair Versus Reconstruction [14]	Ferreira 2022	Retrospective cohort study	III
ACL Repair with Suture Tape Augmentation of Proximal Tears and Early ACL Reconstruction with Suture Tape Augmentation Result in Comparable Clinical Outcomes to ACL Reconstruction at 2‐year Follow‐up [41]	Simard 2024	Retrospective cohort study	III

*Note*: This table lists the title, authors, publication year, study design and levels of evidence [9] for studies included in this systematic review.

Abbreviation: ACL, anterior cruciate ligament.

### Methodological quality and risk of bias assessment

Overall, the included studies assessed using the ROBINS‐I tool showed different levels of risk of bias in different domains. As the study aimed to describe the results for a single group of patients undergoing ACL repair, confounding bias (D1) was not deemed relevant and was categorized as ‘Not Applicable’. Most articles showed low risk in areas such as selection of participants, but several showed moderate or serious risk in areas related to missing data and measurement of outcomes (D5 and D6).

**Figure 2:**
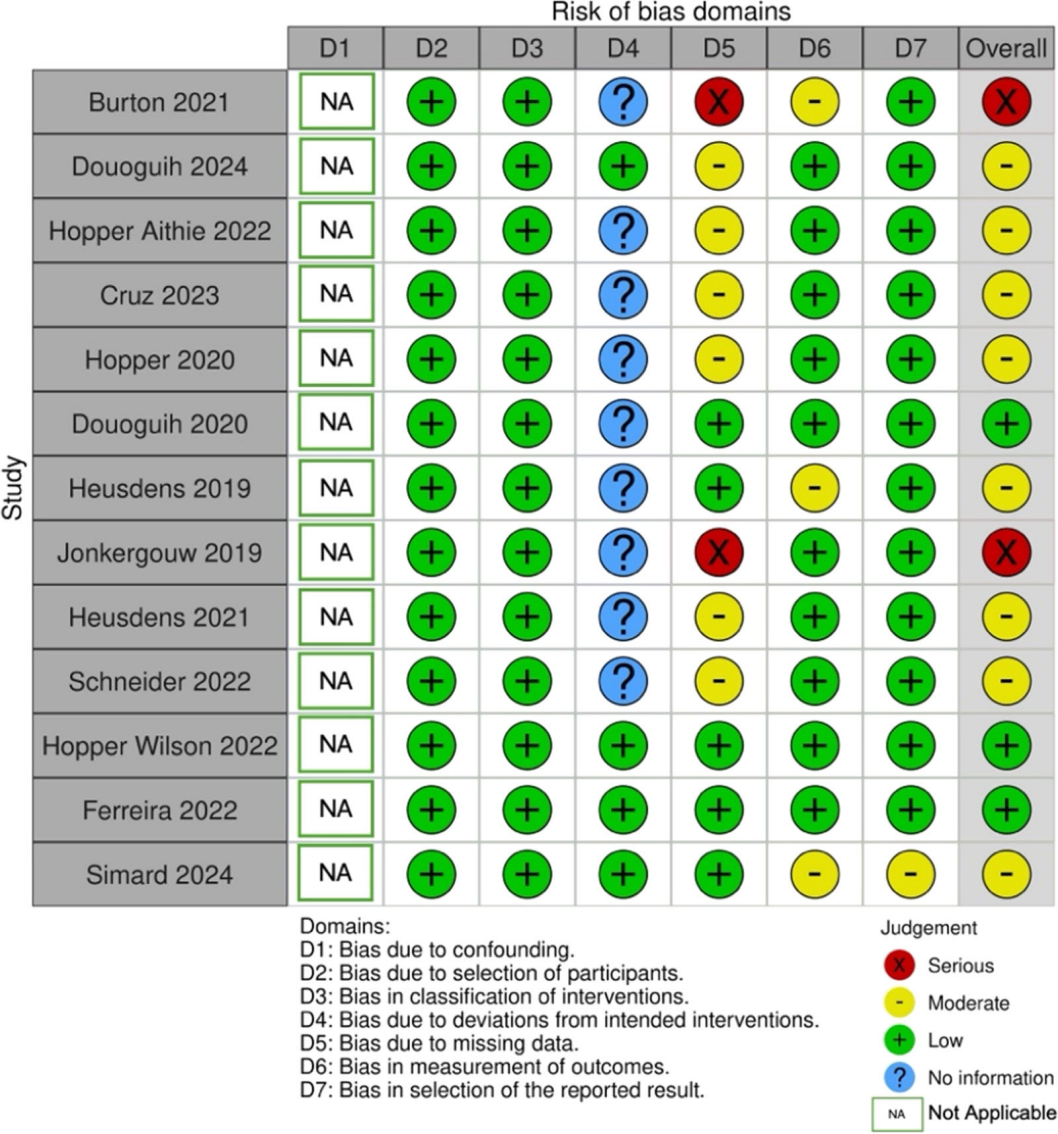
Risk of bias assessment for included studies. This figure summarizes the risk of bias across seven domains (D1–D7) for studies included in the systematic review, assessed using the ROBINS‐I tool [42]. The judgement categories include low risk (green), moderate risk (yellow), serious risk (red), and no information available (blue) or not applicable (NA). Overall risk of bias is presented in the final column. ROBINS‐I, Risk of Bias in Non‐randomized Studies‐Interventions.

Risk of bias assessment is displayed in Figure 2.

### Demographic data

A total of 671 patients undergoing primary ACL repair augmented with internal brace were included in the review. The mean age of all patients was between 25.7 and 43.2 years, and the mean follow‐up was between 2 and 5.7 years. Study characteristics and patient demographics extracted from the included studies are shown in Table 2.

**Table 2:** Patient demographics and follow‐up in the studies investigating suture‐augmented (SA) primary ACL repair.

First author year	Follow‐up period in years, mean ± SD	Patients receiving SA ACL Repair, *n*	Age, mean ± SD	Lost to follow‐up, %	Female, %
Burton 2021	2.8 ± 0.9	35	32.2 ± 7.2	17.1%	40.0%
Douoguih 2024	Minimum of 2 years	30	27.5 ± n.r.	13.3%	56.7%
Hopper Aithie 2022	5.7 ± 0.5	37	37.8 ± 15.5	8.1%	47.1%
Cruz 2023	3.1 ± 1.1	52	28.3 ± 8.4	11.5%	30.4%
Hopper 2020	3.7 ± 0.8	43	25.7 ± 10.1	11.6%	44.7%
Douoguih 2020	2.8 ± 0.7	28	27.4 ± 8.6	3.6%	33.3%
Heusdens 2019	2.0 ± n.r.	43	33 ± 14.5	2.3%	42.9%
Jonkergouw 2019	2.4 ± 0.7	27	29.6 ± 10.1	n.r.	44.4%
Heusdens 2021	2.0 ± n.r.	37	32.8 ± 9.7	5.4%	51.4%
Schneider 2022	3.0 ± n.r.	93	n.r.	7.5%	72.0%
Hopper Wilson 2022	5.0 ± n.r.	137	35.0 ± 14.0	2.2%	44.0%
Ferreira 2022	2.5 ± 0.4	75	40.0 ± 11.0	0.0%	61.3%
Simard 2024	2.0 ± n.r.	34	43.2 ± 9.9	0.0%	51.7%

Abbreviations: ACL, anterior cruciate ligament; SD, standard deviation.

### Preoperative PROMs

Patients reported a mean IKDC of 49.5 (41.7–57.3) and Lysholm score of 52.7 (43.8–61.7). KOOS subscale scores included a mean KOOS Total of 49.5 (44.9–54.0), KOOS Pain of 64.6 (62.1–67.1), KOOS Symptoms of 57.7 (52.0–63.5), KOOS ADL of 70.3 (63.4–77.3), KOOS Sport of 27.2 (20.4–34.1) and KOOS QoL of 26.7 (24.2–29.1). The mean VAS for pain was 2.9 (2.5–3.2). Activity levels resulted in a mean MARX score of 11.7 (9.9–13.6), SANE score of 40.4 (20.3–60.6) and Tegner score of 5.5 (2.4–8.5). WOMAC subscale scores included a mean WOMAC Pain of 77.1 (72.8–81.40), WOMAC Function of 74.7 (70.3–79.0) and WOMAC Stiffness of 65.5 (59.9–71.2). The mean preoperative side‐to‐side difference measured by KT‐1000 was 2.7 mm (0.8–4.6). The average values of the reported preoperative PROMs are presented in Table 3 (and Figures A1–A14 in the Appendix S2).

**Table 3:** Mean preoperative scores and corresponding 95% confidence intervals for each reported outcome measure across the included studies.

Outcome measure	Mean score	95% Confidence Interval
IKDC	49.5	41.67−57.33
KOOS Pain	64.58	62.05−67.10
KOOS Symptoms	57.71	51.96−63.46
KOOS ADL	70.32	63.36−77.29
KOOS Sport	27.22	20.40−34.05
KOOS QoL	26.66	24.19−29.13
KOOS Total	49.47	44.91−54.03
VAS Pain	2.88	2.52−3.24
MARX	11.73	9.85−13.62
SANE	40.43	20.26−60.60
Tegner	5.48	2.44−8.52
Lysholm	52.7	43.75−61.65
WOMAC Pain	77.10	72.76−81.44
WOMAC Function	74.65	70.30−79.01
WOMAC Stiffness	65.53	59.86−71.19
KT‐1000 (mm)	2.72	0.84−4.61

Abbreviations: IKDC, International Knee Documentation Committee; KOOS, Knee injury and Osteoarthritis Outcome Score; QoL, quality of life; SANE, single assessment numeric evaluation; VAS, visual analogue scale; WOMAC, Western Ontario and McMaster Universities Osteoarthritis Index.

### ACL re‐rupture, reoperations and complications

Details on reruptures and reoperations are reported in Table 4.

**Table 4:** Re‐rupture rates and non‐rupture‐related re‐operations in patients undergoing suture‐augmented ACL repair.

First author, year	Re‐rupture rate, *n* (%)	Age at re‐rupture, mean ± SD	Timing of re‐rupture, mean ± SD	Re‐operations not related to ACL re‐rupture, *n* (%)	Re‐operations not related to ACL re‐rupture, description
Burton 2021	2 (6.9%)	n.r.	1.1 ± 0.6	n.r.	n.r.
Douoguih 2024	3 (11.5%)	19.6 ± 4.67	1.1 ± 0.3	n.r.	n.r.
Hopper Aithie 2022	6 (17.6%)	20.7 ± n.r.	n.r.	3 (8.8%)	Secondary meniscectomies
Cruz 2023	12 (26.1%)	n.r.	n.r.	0	n.a.
Hopper 2020	2 (5.3%)	22.0 ± 11.3	n.r.	0	n.a.
Douoguih 2020	4 (14.8%)	n.r.	n.r.	0	n.a.
Heusdens 2019	2 (4.8%)	n.r.	0.8 ± 0.6	n.r.	n.r.
Jonkergouw 2019	2 (7.4%)	n.r.	1.2 ± 0.2	2 (7.4%)	2 tibial anchor removal
Heusdens 2021	4 (11.4%)	n.r.	1.1 ± 0.6	3 (8.6%)	1 medial meniscal rupture; 1 cyclops; 1 excessive tight fibre tape
Schneider 2022	9 (10.0%)	n.r.	1.2 ± 1.2	n.r.	n.r.
Hopper Wilson 2022	22 (16.4%)	n.r.	n.r.	8 (6.0%)	Three ipsilateral partial medial meniscectomies; two ipsilateral partial lateral meniscectomies; two MUA; one chondroplasty
Ferreira 2022	4 (5.3%)	26.8 ± n.r.	n.r.	3 (4.0%)	Hardware removal
Simard 2024	2 (5.9%)	n.r.	n.r.	n.r.	n.r.

Abbreviations: ACL, anterior cruciate ligament; MUA, mobilization under anaesthesia; SD, standard deviation.

**Figure 3:**
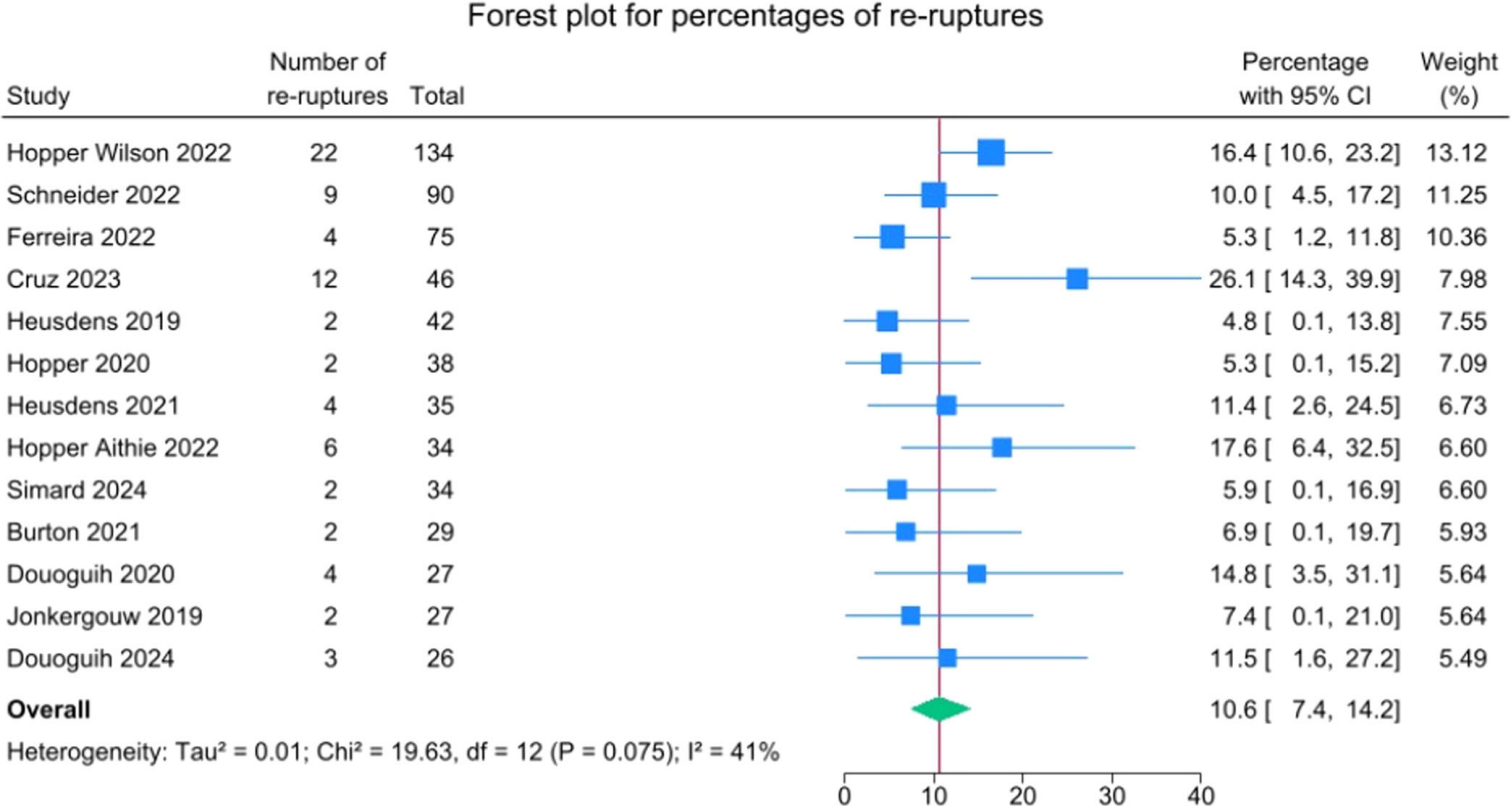
Forest plot of re‐rupture rates in patients undergoing suture‐augmented primary ACL repair. ACL, anterior cruciate ligament; CI, confidence interval.

Overall, 74 patients had their ACL re‐rupture, representing 10.6% (95% CI: 7.4–14.2) of cases across all studies (range reported, 4.8%–26.1%), as shown in Figure 3.

**Figure 4:**
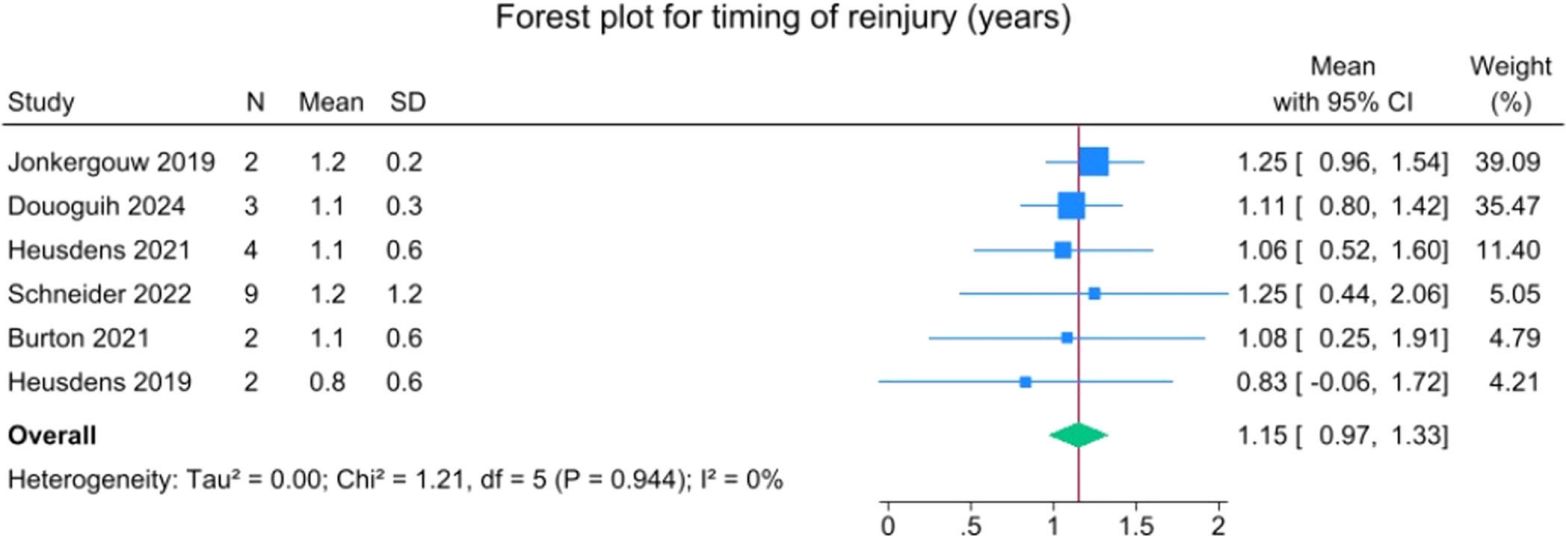
Forest plot of time to reinjury after suture‐augmented primary ACL repair. ACL, anterior cruciate ligament; CI, confidence Interval; SD, standard deviation.

**Figure 5:**
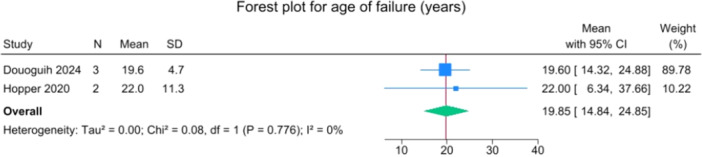
Forest plot of age when the suture‐augmented primary ACL repair failed. ACL, anterior cruciate ligament; CI, confidence Interval; SD, standard deviation.

The overall mean time to reinjury was 1.2 years (1.0–1.3) and at a mean age of 19.8 years (14.8–24.8) (Figures 4 and 5).

**Figure 6:**
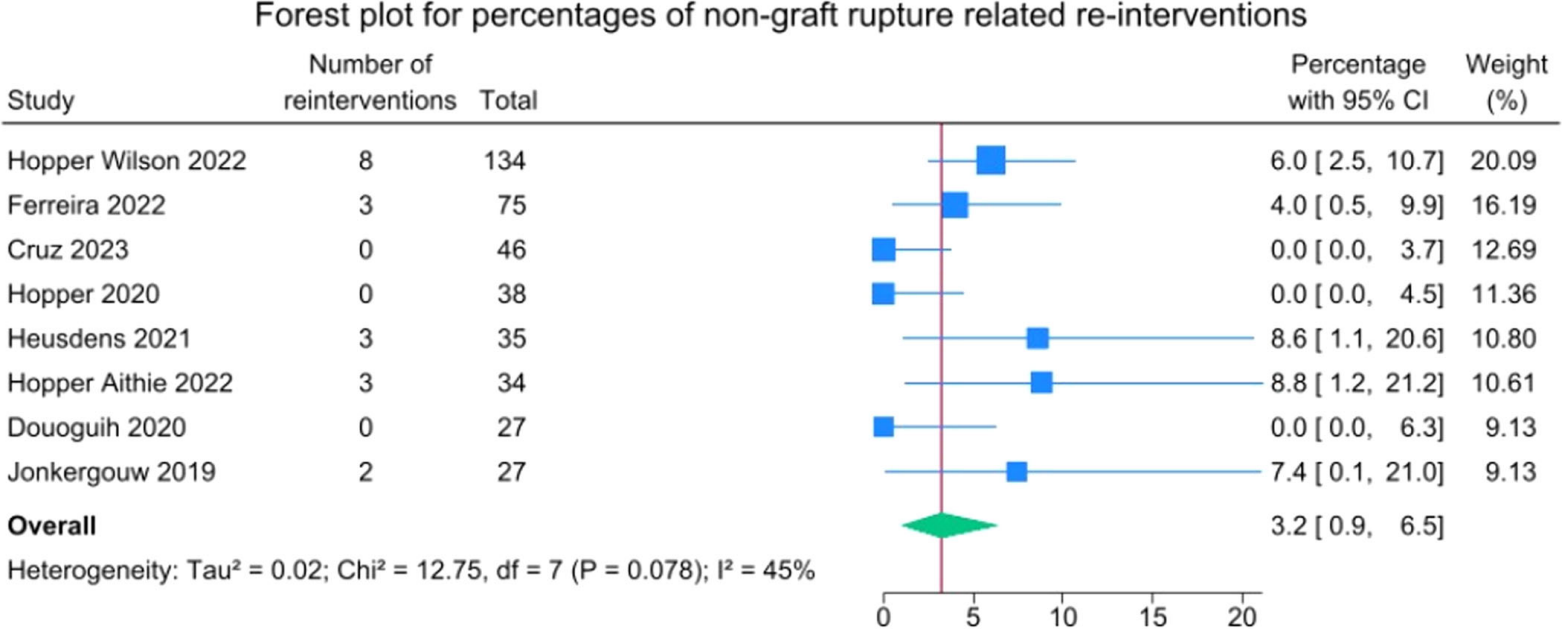
Forest plot of non‐graft rupture‐related complications requiring surgery in suture‐augmented primary ACL repair. ACL, anterior cruciate ligament; CI, confidence Interval; SD, standard deviation.

Reoperation for reasons unrelated to ACL rupture (primarily hardware removal) was performed in 3.2% (0.9–6.5) of patients (Figure 6).

Only one study reported non‐graft rupture‐related complications that did not require surgery, involving 9 out of 75 patients [14]. There was one case of anterior knee pain, seven cases of symptomatic hardware and one case of dysesthesia.

**Figure 7:**
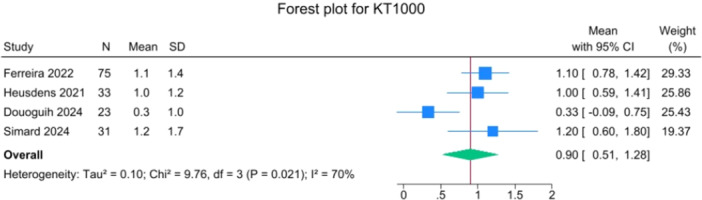
Forest plot of side‐to‐side laximetry, in mm, after suture‐augmented primary ACL repair. ACL, anterior cruciate ligament; CI, confidence interval.

Data from arthrometer side‐to‐side comparisons were reported for 162 patients. The mean of the reported side‐to‐side laxity was 0.90 mm (0.5–1.3), with a range of 0.3–1.2 mm (Figure 7).

### Post‐operative PROMs and RTS

**Figure 8:**
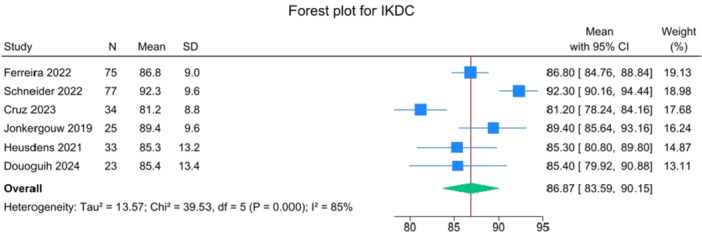
Forest plots showing the distribution of post‐operative IKDC across the studies. CI, confidence interval; IKDC, International Knee Documentation Committee.

**Figure 9:**
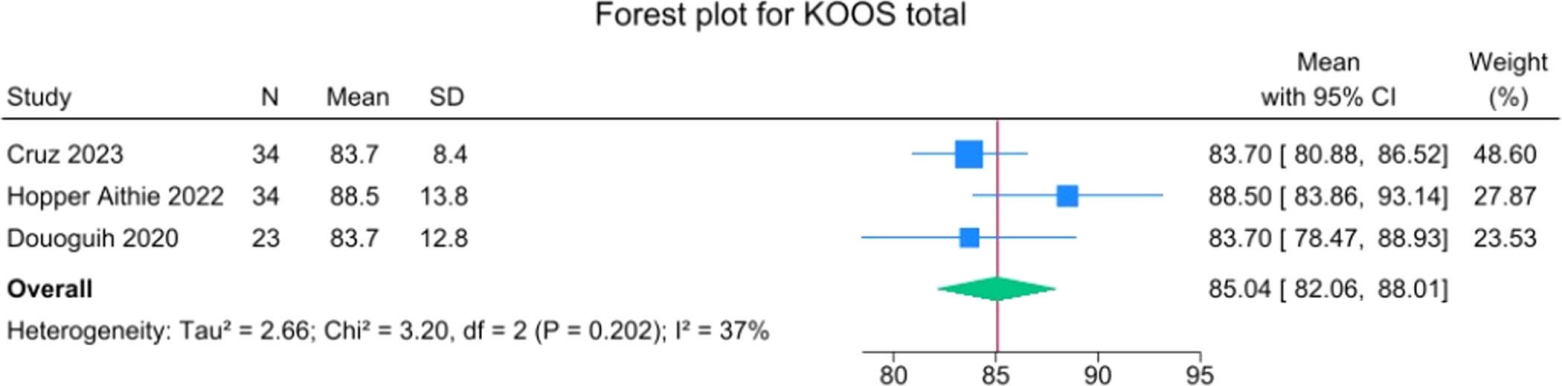
Forest plots showing the distribution of post‐operative KOOS across the studies. CI, confidence interval; KOOS, Knee injury and Osteoarthritis Outcome Score.

**Figure 10:**
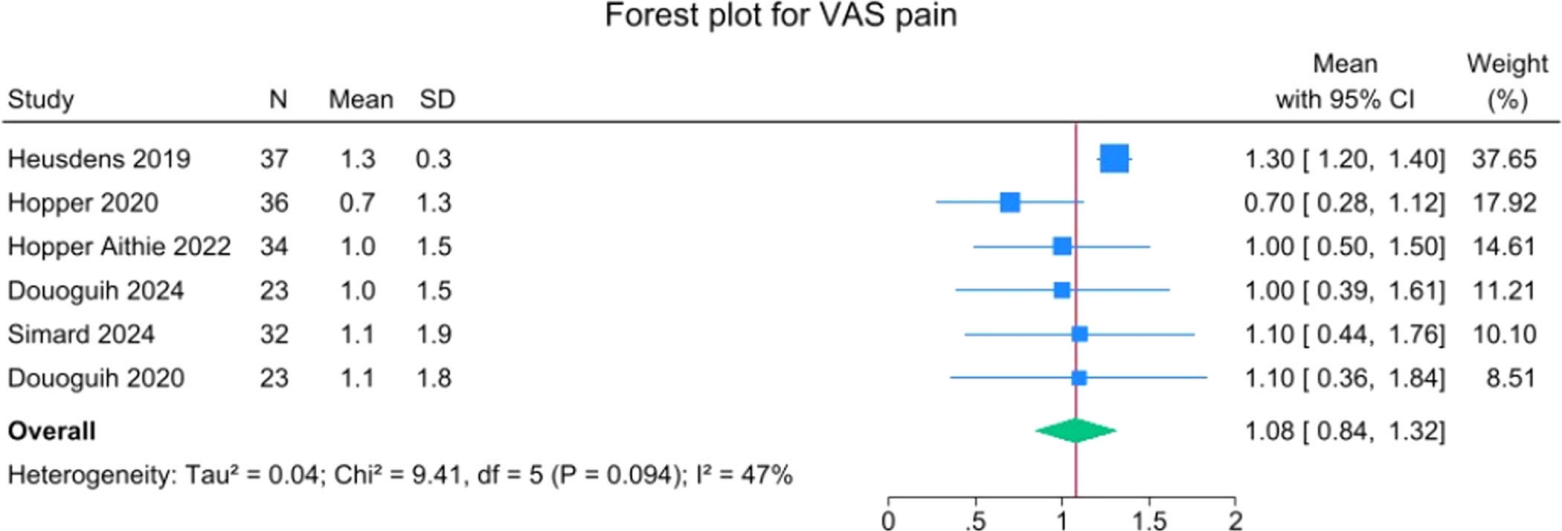
Forest plots showing the distribution of post‐operative VAS pain across the studies. CI, confidence interval; VAS, visual analogue scale.

**Figure 11:**
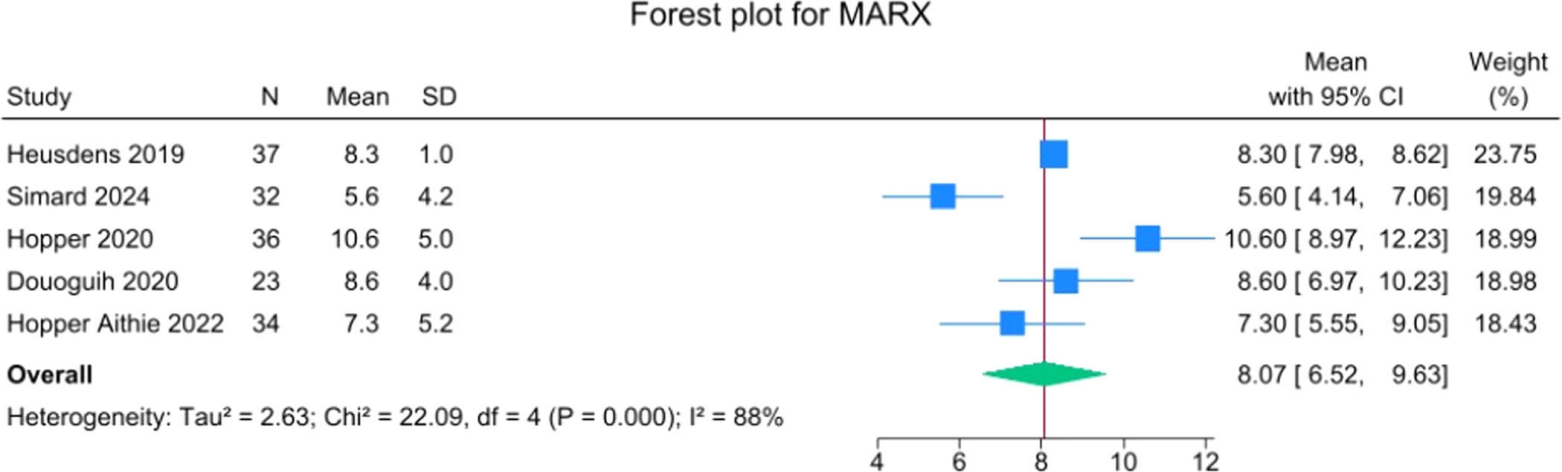
Forest plots showing the distribution of post‐operative MARX across the studies. CI, confidence interval.

**Figure 12:**
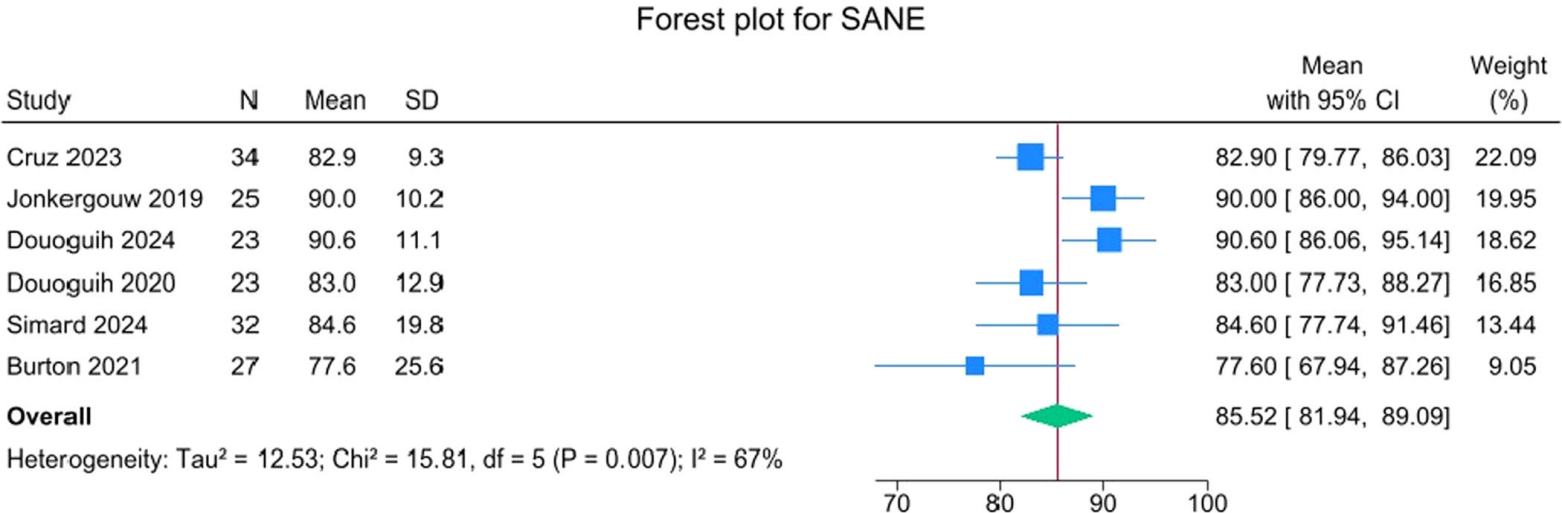
Forest plots showing the distribution of post‐operative SANE across the studies. CI, confidence interval.

**Figure 13:**
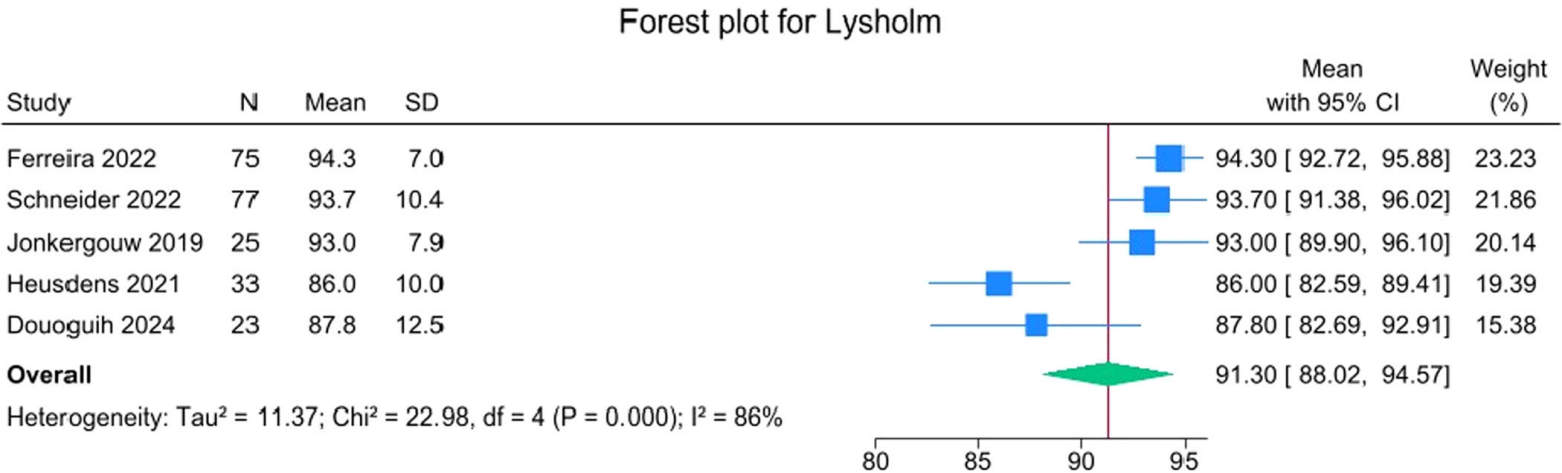
Forest plots showing the distribution of post‐operative Lysholm across the studies. CI, confidence interval.

**Figure 14:**
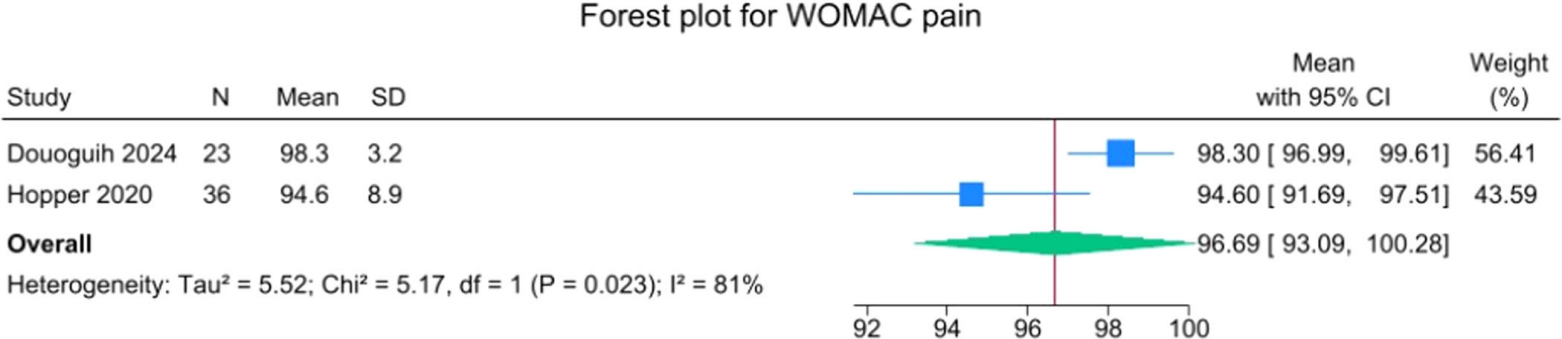
Forest plots showing the distribution of post‐operative WOMAC pain across the studies. CI, confidence interval.

**Figure 15:**
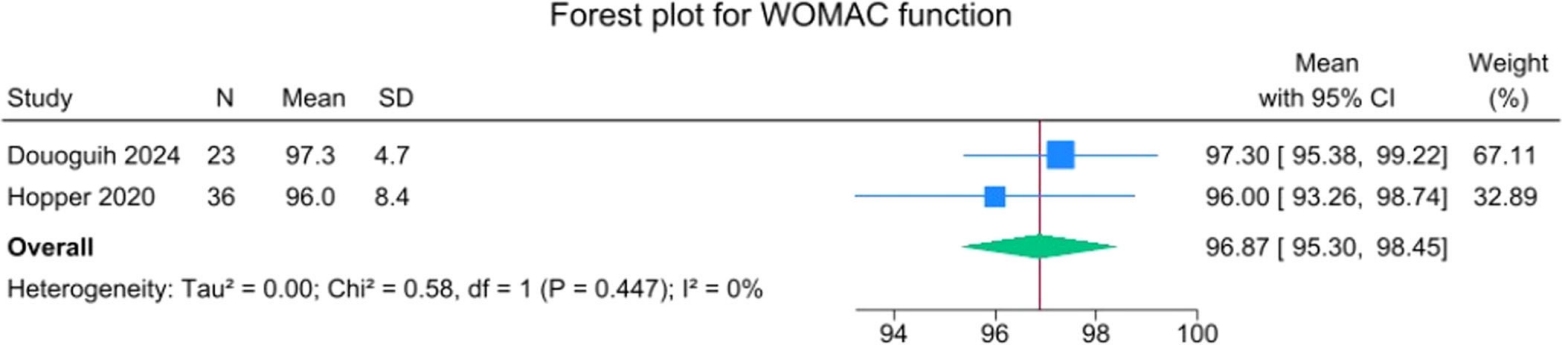
Forest plots showing the distribution of post‐operative WOMAC fucntion across the studies. CI, confidence interval.

**Figure 16:**
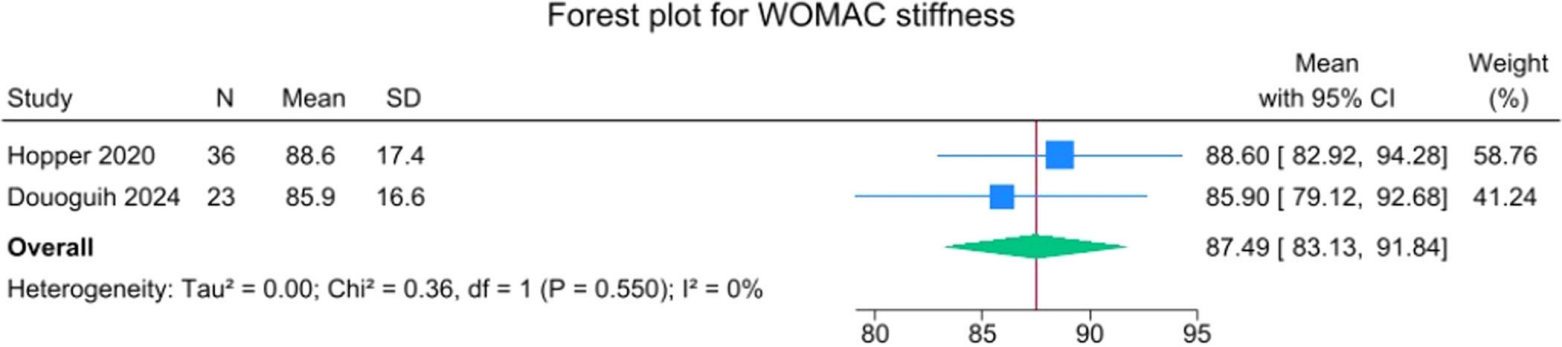
Forest plots showing the distribution of post‐operative WOMAC stiffness across the studies. CI, confidence interval.

Patients reported a mean IKDC of 86.9 (83.6–90.2), KOOS Total of 85.0 (82.1–88.0), WOMAC Function of 96.9 (95.3–98.4), WOMAC Stiffness of 87.5 (83.1–91.8) and Lysholm of 91.3 (88.0–94.6). The mean VAS for pain was 1.1 (0.8–1.3), and the WOMAC Pain was 96.7 (93.1–100.3). Activity levels resulted in a mean MARX score of 8.1 (6.5–9.6) and a SANE score of 85.5 (81.9–89.1). The average values of the reported PROMs are presented in Figures 8, 9, 10, 11, 12, 13, 14, 15, 16.

**Figure 17:**
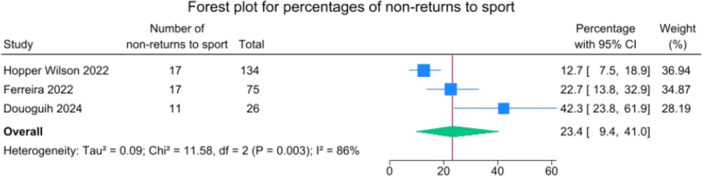
Forest plot of non‐return to sport rates in patients undergoing suture‐augmented primary ACL repair. ACL, anterior cruciate ligament; CI, confidence interval.

Three studies provided data on RTS rates. Out of 235 patients, 190 successfully returned to sports, with an overall rate of 76.6% (59.0–90.6). RTS rates ranged from 57.7% to 87.3% (Figure 17).

**Figure 18:**
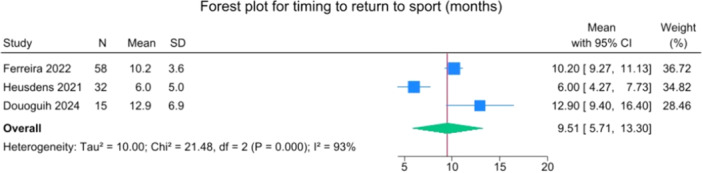
Forest plot of time to return to sport. CI, confidence interval; SD, standard deviation.

**Figure 19:**
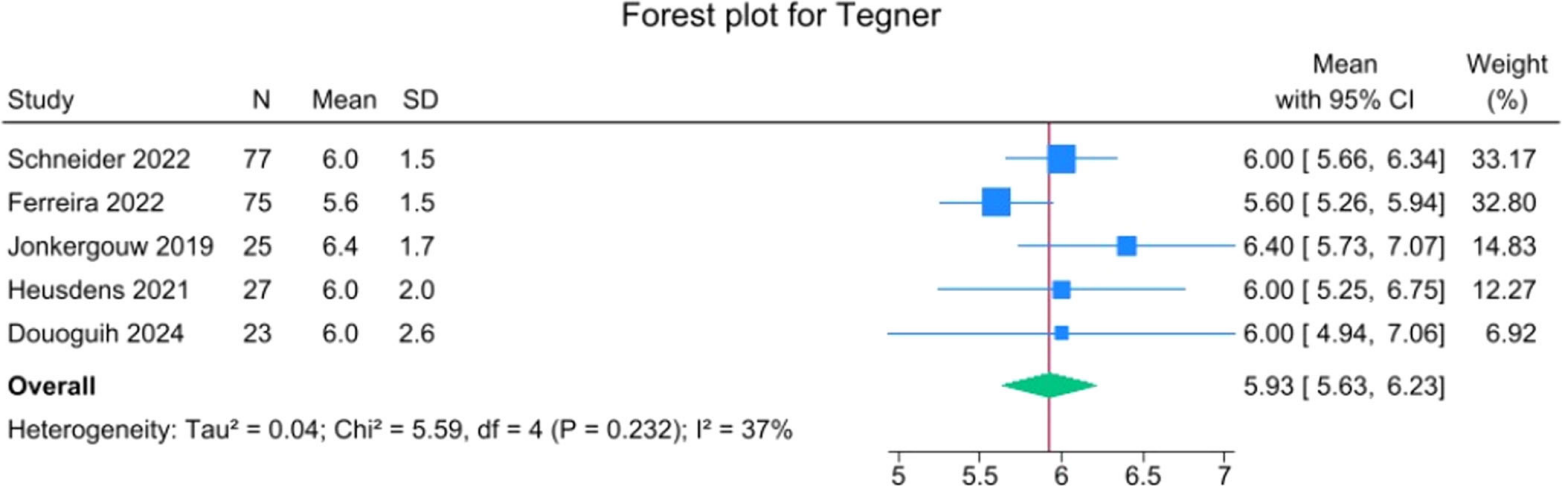
Forest plot of post‐operative Tegner activity scale. CI, confidence interval SD, standard deviation.

Specific data on return to pre‐injury activity levels were provided by Heusdens et al. and Ferreira et al. [14, 21]. In these two studies, 74 out of 110 patients returned to their pre‐injury activity level, or 67.3%, with reported rates ranging from 66.7% to 74.7%. The overall mean for time to RTS was 9.5 months (5.7–13.3), and the mean Tegner activity scale was 5.9 (5.6–6.2) (Figures 18 and 19).

## DISCUSSION

The main finding of this study was that SA primary ACL repair has satisfactory clinical outcomes, functional outcomes and moderately low re‐rupture rates without a high number of specific complications. In the current review, the overall re‐rupture rate was approximately 11% (range, 4.8%–26.1%), despite the inclusion of studies with heterogeneous populations and varying risk factors for re‐rupture. These failure rates are consistent with those observed with the gold‐standard ACL reconstruction techniques for the treatment of ACL injuries [19, 20, 27, 36]. Historically, the main concern with ACL primary repair has been the high re‐rupture rates. These studies, which were conducted before arthroscopic surgery became the gold standard, have typically included heterogeneous cohorts, featuring a wide range of age groups and patients with concomitant knee injuries, and did not differentiate based on tear pattern or location [1, 11, 13, 15, 30].

The use of internal bracing in conjunction with primary ACL repair could offer some benefits, as this ligament augmentation technique aims to stabilize and reinforce the ligament, protect the tissue during the healing process, while allowing early mobilization [32]. Still, since this is not a comparative study, the beneficial effect of internal bracing in addition to primary ACL repair cannot be directly inferred from the current study.

The age range of the included patients was wide, with a mean age of 26–43 years. Age is an important determinant of ACL reparability, with the likelihood of successful repair increasing in older patients. In fact, van der List et al. conducted a retrospective case–control study searching for predictive factors for the possibility of arthroscopic primary ACL repair [31]. They included 361 patients, of whom ACL primary repair was possible in 158 patients. Multivariate analysis showed that age >35 years, surgery within 28 days, and BMI < 26 were predictive of the possibility of primary repair. Even Vermeijden et al., who investigated predictors of ACL tear patterns in a cohort of 254 patients, could not identify anatomic risk factors as having a role in tear location but found that proximal ACL tears were more commonly found in older patients [44].

Primary ACL repair appears to lead to favourable outcomes for returning to sports. In this review, the RTS outcomes following SA primary ACL repair were encouraging. In the studies reporting RTS data, over 75% of patients successfully returned to sport, with rates up to 87%. Also, 67% of patients returned to their pre‐injury activity level; the average time to RTS was 9.5 months, and the mean post‐operative Tegner activity score was 5.9. Similarly, Annibaldi et al. recently reported a 74% RTS rate and a 93.5% return to pre‐injury level in a cohort of soccer players following ACL repair [2].

PROMs following SA primary ACL repair were favourable. The mean post‐operative IKDC and KOOS total scores were 86.9 and 85.0, respectively, and high scores were observed for WOMAC Function (96.9), WOMAC Stiffness (87.5), and Lysholm (91.3). Pain levels were low, with a mean VAS score of 1.1 and WOMAC Pain of 96.7. Activity‐related outcomes were also positive, with MARX and SANE scores averaging 8.1 and 85.5, respectively. These results are supported by comparative studies. For example, Vermeijden et al. compared patients who underwent ACL repair in one knee and ACL reconstruction in the other knee. They reported no significant differences in functional scores, but notably, many patients preferred the repaired knee due to less pain, an earlier return of range of motion, and faster rehabilitation [43]. Ferretti et al. found that ACL repair combined with anterolateral structure repair achieved PROMs comparable to ACL reconstruction plus LET, but with better forgotten joint score (FJS‐12) and a higher share of patients reaching the patient‐acceptable symptom state (PASS) threshold for KOOS subdomains [16]. Similarly, Ferreira et al. reported superior FJS‐12 scores and faster hamstring strength recovery in the repair group, with a higher proportion reaching the PASS threshold [14]. In a recent series of acute primary ACL repairs for Sherman type I–II tears, Monaco et al. reported progressive magnetic resonance imaging evidence of ligament maturation over the first post‐operative year and favourable 2‐year functional outcomes, with more than 86% of patients achieving PASS [35]. These findings reinforce the idea that primary ACL repair, when indicated appropriately, can achieve PROMs that are at least comparable to those of reconstruction, with potential benefits in terms of joint perception and functional recovery.

This systematic review has limitations. First, the inclusion of case series, which provides the lowest LoE. Also, the included studies varied significantly in design, from case series to comparative prospective cohort studies. This heterogeneity in study design and methodological rigour limits the generalizability of our findings and means that our results should be interpreted cautiously. Additionally, the follow‐up in the included studies, while meeting the minimum 2‐year requirement, may not be sufficient to reveal failures or long‐term complications. The relatively high re‐rupture rates reported in some studies, such as 26.1% in the study by Cruz et al., also highlight the need for other investigations on the factors contributing to failure of ACL primary repair, including patient selection, surgical technique and rehabilitation protocols.

This systematic review has several strengths. First, it adheres to rigorous methodological standards by following the PRISMA guidelines and with a registered protocol in the PROSPERO database, which increases transparency and reduces the risk of selective reporting. Second, it uses a comprehensive search strategy that includes the PubMed, Embase and Cochrane Library databases, which have a wide range of potentially relevant studies. Third, the authors included only studies with at least 2 years of follow‐up and report both re‐rupture rates and validated PROMs (e.g., IKDC, KOOS, Lysholm or Tegner scales), providing a clinically meaningful assessment of safety and efficacy over time. Finally, the large overall sample size (671 patients) and the use of quantitative synthesis, including the calculation of overall re‐rupture rates and functional outcomes, allow for an understanding of the advantages and disadvantages of SA primary ACL repair with internal brace.

## CONCLUSIONS

SA primary ACL repair with internal brace shows acceptable clinical results, encouraging functional recovery, and re‐rupture rates. Internal bracing is not associated with a high rate of complications, suggesting that the technique is safe and appropriate for use in carefully selected patients. However, heterogeneity in the existing studies means that additional high‐quality, long‐term comparative studies are needed to clarify the indications and reliability of this procedure.

## AUTHOR CONTRIBUTIONS

All authors contributed to the study. Etienne Cavaignac and Edoardo Monaco have ideated the study and established the study design. Material preparation, data collection and analysis were performed by Valerio Nasso, Alessandro Carrozzo, Émilie Bérard and Jonathan Rioual. The first draft of the manuscript was written by Alessandro Carrozzo, and Etienne Cavaignac, Edoardo Monaco and Régis Pailhe had substantially edited the draft. All authors read and approved the final manuscript.

## CONFLICT OF INTEREST STATEMENT

Etienne Cavaignac: Consultant for Arthrex, Amplitude and Biobank. Edoardo Monaco: Consultant for Arthrex. The remaining authors declare no conflicts of interest.

## ETHICS STATEMENT

The ethics statement is not available.

## Supporting information

Appendix S1.

Appendix S2.

## Data Availability

The data sets generated and analyzed during the current study are not publicly available but are available from the corresponding author on reasonable request.
