# Supplementary material for: Suture‐augmented primary anterior cruciate ligament repair with internal brace shows acceptable re‐rupture rates, favourable outcomes and high return‐to‐sport rates: A systematic review
Source: J Exp Orthop. 2025 Nov 5;12(4):e70495. doi: 10.1002/jeo2.70495 (PMC12588170; doi:10.1002/jeo2.70495)
Supplement: Supplementary file 1 — Appendix S1. [file JEO2-12-e70495-s002.docx]

Appendix 1

Pubmed accessed on august 30, 2024

(("Anterior Cruciate Ligament"[Mesh] OR "anterior cruciate ligament"[tiab] OR ACL[tiab])

AND

("Primary Repair"[tiab] OR "Ligament Repair"[tiab] OR repair[tiab] OR "internal brace"[tiab] OR "internal bracing"[tiab] OR "suture augmentation"[tiab] OR augmentation[tiab]))

Filters: English language, Humans

Embase accessed on august 30, 2024

('anterior cruciate ligament'/exp OR 'anterior cruciate ligament':ti,ab OR ACL:ti,ab)

AND

('primary repair':ti,ab OR 'ligament repair':ti,ab OR repair:ti,ab OR 'internal brace':ti,ab OR 'internal bracing':ti,ab OR 'suture augmentation':ti,ab OR augmentation:ti,ab)

AND

[english]/lim AND [humans]/lim

Cochrane library accessed on august 30, 2024

([mh "Anterior Cruciate Ligament"] OR "anterior cruciate ligament":ti,ab,kw OR ACL:ti,ab,kw)

AND

("primary repair":ti,ab,kw OR "ligament repair":ti,ab,kw OR repair:ti,ab,kw OR "internal brace":ti,ab,kw OR "internal bracing":ti,ab,kw OR "suture augmentation":ti,ab,kw OR augmentation:ti,ab,kw)
