# Supplementary material for: Suture‐augmented primary anterior cruciate ligament repair with internal brace shows acceptable re‐rupture rates, favourable outcomes and high return‐to‐sport rates: A systematic review
Source: J Exp Orthop. 2025 Nov 5;12(4):e70495. doi: 10.1002/jeo2.70495 (PMC12588170; doi:10.1002/jeo2.70495)
Supplement: Supplementary file 2 — Appendix S2. [file JEO2-12-e70495-s001.docx]

# Description of preoperative PROMs

## IKDC

Only one study (Douoguih 2024) reported preoperative IKDC score. Among the 29 patients with available data, the mean IKDC score was 49.5 (SD: 21.5; 95% CI: 41.67, 57.33).

## KOOS pain


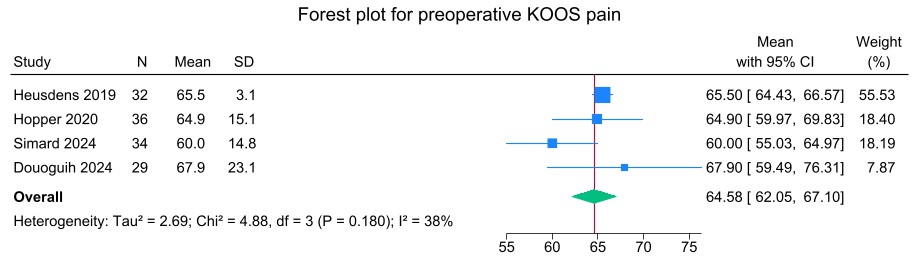


***Forestplot_Mmean_KOOS_pain_preop.pdf***

The overall mean for preoperatively KOOS pain is 64.58 [95% CI: 62.05, 67.10].

These studies exhibit moderate heterogeneity for this measure (I²=38%).

## KOOS symptom


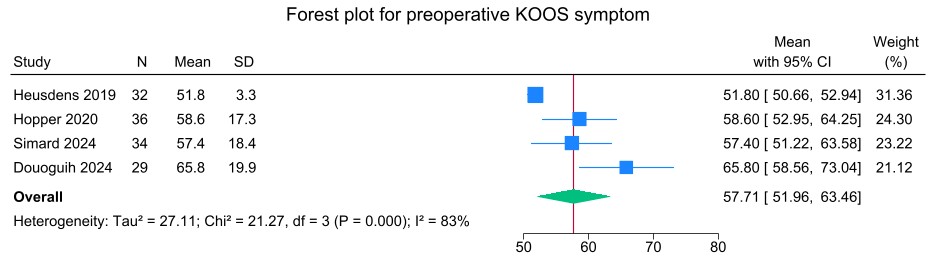


***Forestplot_Mmean_KOOS_symptom_preop.pdf***

The overall mean for preoperatively KOOS symptom is 57.71 [95% CI: 51.96, 63.46].

These studies exhibit high heterogeneity for this measure (I²=83%).

## KOOS activities of daily


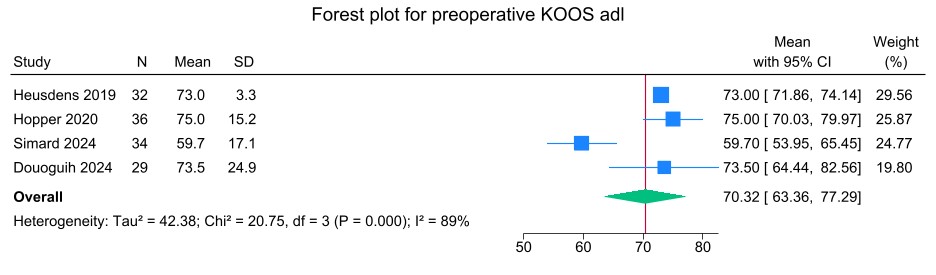


***Forestplot_Mmean_KOOS_adl_preop.pdf***

The overall mean for preoperatively KOOS adl is 70.32 [95% CI: 63.36, 77.29].

These studies exhibit high heterogeneity for this measure (I²=89%).

## KOOS sport


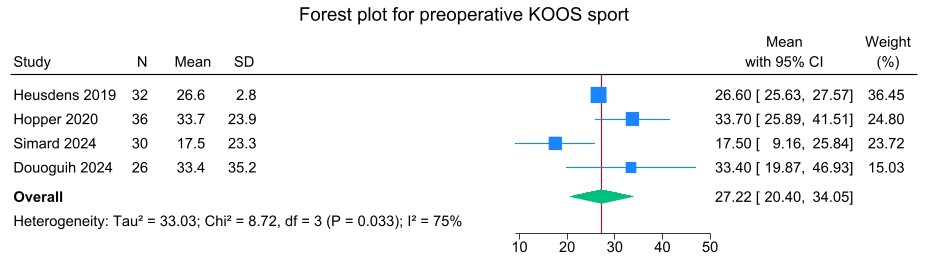


***Forestplot_Mmean_KOOS_sport_preop.pdf***

The overall mean for preoperatively KOOS sport is 27.22 [95% CI: 20.40, 34.05].

These studies exhibit high heterogeneity for this measure (I²=75%).

## KOOS quality of life


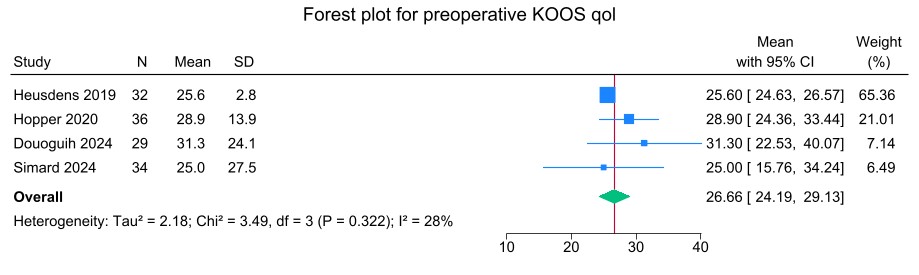


***Forestplot_Mmean_KOOS_qol_preop.pdf***

The overall mean for preoperatively KOOS qol is 26.66 [95% CI: 24.19, 29.13].

These studies exhibit moderate heterogeneity for this measure (I²=28%).

## KOOS total


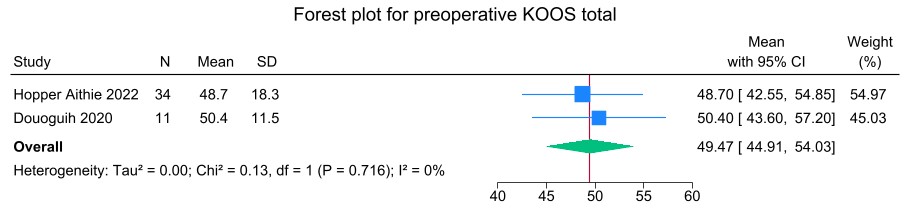


***Forestplot_Mmean_KOOS_total_preop.pdf***

The overall mean for preoperatively KOOS total is 49.47 [95% CI: 44.91, 54.03].

These studies exhibit no heterogeneity for this measure (I²=0%).

## VAS pain


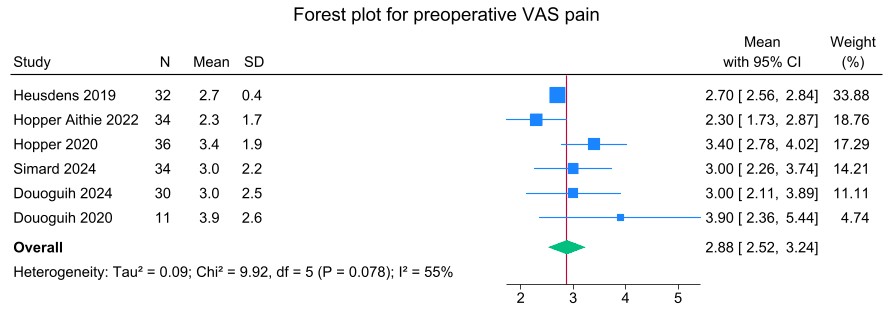


***Forestplot_Mmean_VAS_pain_preop.pdf***

The overall mean for preoperatively VAS pain is 2.88 [95% CI: 2.52, 3.24].

These studies exhibit high heterogeneity for this measure (I²=55%).

## MARX


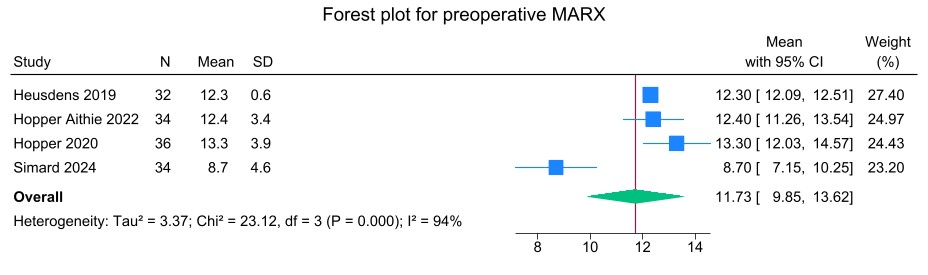


***Forestplot_Mmean_MARX_preop.pdf***

The overall mean for preoperatively MARX is 11.73 [95% CI: 9.85, 13.62].

These studies exhibit high heterogeneity for this measure (I²=94%).

## SANE


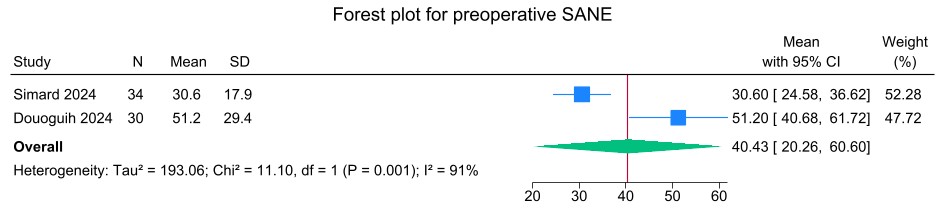


***Forestplot_Mmean_SANE_preop.pdf***

The overall mean for preoperatively SANE is 40.43 [95% CI: 20.26, 60.60].

These studies exhibit high heterogeneity for this measure (I²=91%).

## Tegner


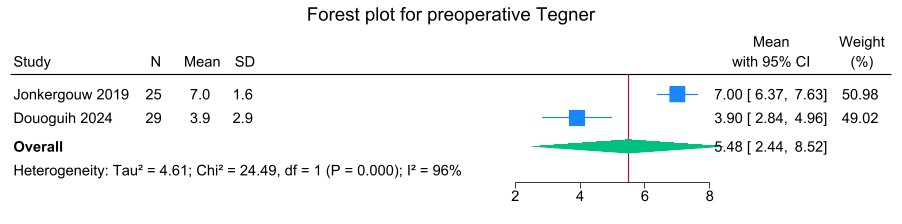


***Forestplot_Mmean_Tegner_preop.pdf***

The overall mean for preoperatively Tegner is 5.48 [95% CI: 2.44, 8.52].

These studies exhibit high heterogeneity for this measure (I²=96%).

## Lysholm

Only one study (Douoguih 2024) reported preoperative Lysholm score. Among the 29 patients with available data, the mean Lysholm score was 52.7 (SD: 24.6; 95% CI: 43.75, 61.65).

## WOMAC pain


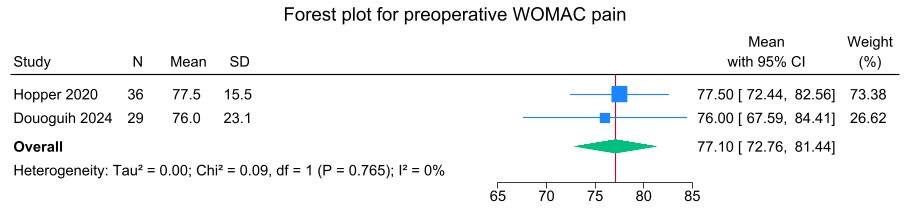


***Forestplot_Mmean_WOMAC_pain_preop.pdf***

The overall mean for preoperatively WOMAC pain is 77.10 [95% CI: 72.76, 81.44].

These studies exhibit no heterogeneity for this measure (I²=0%).

## WOMAC function


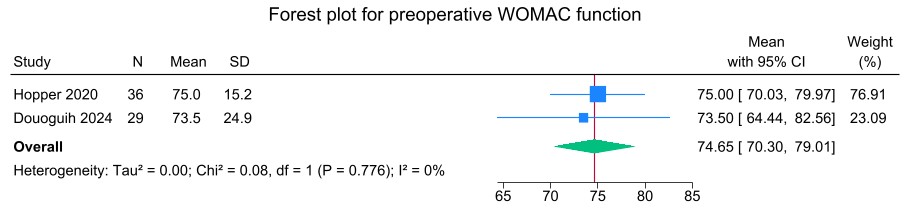


***Forestplot_Mmean_WOMAC_funct_preop.pdf***

The overall mean for preoperatively WOMAC function is 74.65 [95% CI: 70.30, 79.01].

These studies exhibit no heterogeneity for this measure (I²=0%).

## WOMAC stiffness


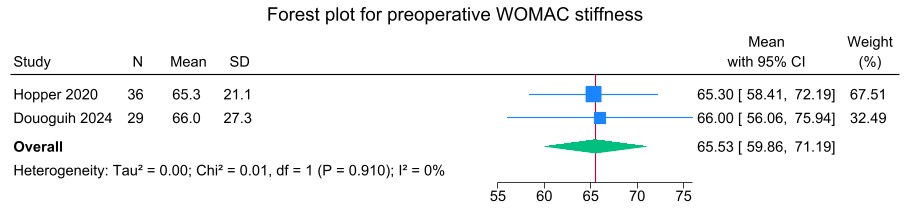


***Forestplot_Mmean_WOMAC_stiff_preop.pdf***

The overall mean for preoperatively WOMAC stiffness is 65.53 [95% CI: 59.86, 71.19].

These studies exhibit no heterogeneity for this outcome measure (I²=0%).

## KT1000


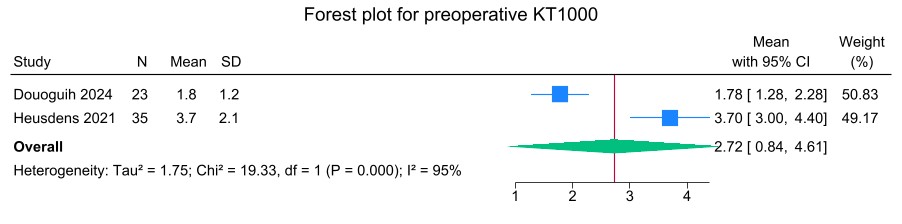


***Forestplot_Mmean_KT1000_preop.pdf***

The overall mean for preoperatively KT1000 is 2.72 [95% CI: 0.84, 4.61].

These studies exhibit high heterogeneity for this outcome measure (I²=95%).
